# The expression of fibrosis-related genes is elevated in doxorubicin-induced senescent human dermal fibroblasts, but their secretome does not trigger a paracrine fibrotic response in non-senescent cells

**DOI:** 10.1007/s10522-022-10013-y

**Published:** 2023-01-17

**Authors:** Fariba Nosrati, Johannes Grillari, Mahnaz Azarnia, Mohammad Nabiuni, Reza Moghadasali, Latifeh Karimzadeh, Ingo Lämmermann

**Affiliations:** 1 Department of Animal Biology, Faculty of Biological Science, Kharazmi University, Tehran, Iran; 2 Department of Biotechnology, Institute of Molecular Biotechnology, University of Natural Resources and Life Sciences, 1190 Vienna, Austria; 3 Ludwig Boltzmann Institute for Traumatology, The Research Center in Cooperation with AUVA, Vienna, Austria; 4 Austrian Cluster for Tissue Regeneration, Vienna, Austria; 5 Department of Cellular and Molecular Biology, Faculty of Biological Sciences, Kharazmi University, Tehran, Iran; 6 Department of Stem Cells and Developmental Biology, Cell Science Research Center, Royan Institute for Stem Cell Biology and Technology, ACECR, Tehran, Iran; 7 Rockfish Bio AG, Vienna, Austria

**Keywords:** Dermal fibrosis, Senescence, Fibroblasts, Fibrotic genes, Aging

## Abstract

Tissue fibrosis is associated with the aging process of most of our organs, and organ aging correlates with the chronic accumulation of senescent cells. Fibrosis occurs when fibroblasts proliferate and deposit pathological amounts of extracellular matrix (ECM), leading to progressive tissue scarring and organ dysfunction. Fibroblasts play a key role in fibrosis, especially in the skin where fibroblasts are the most abundant cell type in the dermis and are mainly responsible for the synthesis of ECM. This study aims to investigate how senescent fibroblasts and their secretome influence dermal fibrosis. Here we used human dermal fibroblasts (HDFs) treated with doxorubicin (doxo) to induce senescence. The senescent phenotype of these stress-induced premature senescent (SIPS) cells was confirmed with several markers. The expression of pro-fibrotic genes was quantified and finally, the impact of their secretome on the fibrotic response of non-senescent fibroblasts was assessed. Doxorubicin treatment, induced senescence in fibroblasts which has been confirmed with elevated senescence-associated β- galactosidase (SA-β-gal) activity, absence of BrdU incorporation, upregulation of p21, and loss of Lamin b1. Expression levels of the pro-fibrotic genes ACTA2 and FN1 increased in SIPS cells, but in contrast to studies using lung fibroblasts the secretome of these cells failed to induce a paracrine fibrotic response in non-senescent cells. In general, these results suggest that these senescent cells are potentially profibrotic, and their accumulation can trigger fibrosis in organs.

## Introduction

Fibrosis is a result of fibroblast activation which is characterized by proliferation, migration, and excessive extracellular matrix deposition and can be triggered by tissue damage and chronic inflammation. In aged organisms the ability for fibrosis to be resolved after wound healing decreases. Resistant fibrosis then can lead to severe chronic diseases (Schafer et al. 2017). Fibrosis is involved in several age-associated diseases, including idiopathic pulmonary fibrosis (IPF), chronic kidney disease (CKD), liver fibrosis, cardiac fibrosis, and skin fibrosis. In developed countries, fibrosis is estimated to be responsible for about 45% of deaths (Jun and Lau 2018). The correlation between fibrosis and accumulation of senescent fibroblast was studied in some of these organs (Schafer et al. 2017; Blokland et al. 2021; Li et al. 2021; Krizhanovsky et al. 2008).

Several types of fibrotic skin tissue exist, from mild, pale, and relatively static atrophic scars to severe highly pigmented, and rapidly growing hypertrophic and keloid scars (Johnson et al. 2020). In developed countries, more than 100 million people are affected by skin fibrosis each year. Fibroblasts as an abundant cell type in the skin dermis have a critical role; they are responsible for synthesizing extracellular matrix during wound healing, and at the same time keeping skin integrity with their senescence when damage occurs (Griffin et al. 2020). In fact, during wound healing, cell senescence plays a dual role; its endogenous induction is essential for the healing process and limits fibrosis. As shown in some studies the genetic clearance of senescent cells or their elimination with senolytics retards the healing process and prolongs wound closure. Still, the persistent presence and accumulation of senescent cells lead to the declining wound healing observed in aged individuals and contribute to many age-associated pathologies like organ fibrosis (Pils et al. 2021; Demaria et al. 2014; Johmura et al. 2021). There is increasing evidence showing the role of fibroblast senescence in skin aging, for example, p16^INK4a^-positive senescence fibroblasts increase in the dermis during aging and their abundance correlates with the formation of wrinkles in human skin (Waaijer et al. 2016). Fibroblast senescence is a permanent cell cycle exit state which drives skin aging, general aging, and aging-related disease (Wlaschek et al. 2021).

In general, a permanent arrest in the cell cycle caused by various cellular stresses, regardless of existing growth-inducing agents, is known as cellular senescence. Quiescence, on the other hand, is a temporary state of cell cycle arrest as a result of a lack of growth stimulation or contact inhibition (Valentijn et al. 2018; Blagosklonny 2011). Many stimuli can drive cells into cellular senescence, including DNA damage, telomeres shortening, oncogenic proteins, fatty acids, reactive oxygen species, mitogens, and cytokines, involving pathways such as p16/Rb (retinoblastoma), p53/p21, and possibly others (Wang et al. 2021).

Additionally, senescent cells exhibit altered chromatin organization and gene expression. As a result of these changes, numerous pro-inflammatory cytokines, chemokines, growth factors, proteases, lipid mediators, and extracellular vesicles are secreted, a phenomenon called senescence-associated secretory phenotype (SASP)(Campisi 2013; Narzt et al. 2021; Terlecki-Zaniewicz et al. 2018). Through this SASP, senescent cells affect neighboring cells, negatively influence the tissue microenvironment and promote organ fibrosis (Coppé et al. 2010).

Doxorubicin is a common chemotherapeutic drug and a potent DNA-damaging agent which can induce senescence in vivo and in vitro (Baar et al. 2017; Lee et al. 2019). In this study, we used doxorubicin to induce senescence in human dermal fibroblast, which we confirmed with several senescence markers. To elucidate the role of cellular senescence in the development of fibrosis, we evaluated mRNA levels of fibrotic genes in these senescent fibroblasts and investigated if conditioned media from senescent HDFs induces a fibrotic response in non-senescent cells. The results presented here suggest that although the fibrosis-related genes are upregulated in senescent HDFs their secretome does not induce a paracrine fibrotic response in non-senescent cells which is in contrast to studies in lung fibroblasts (Schafer et al. 2017).

## Materials and methods

### Cell culture

Human dermal fibroblasts isolated from skin biopsies of healthy female donors between the ages of 49 and 65 were obtained from Evercyte (Vienna, Austria). The HDFs were cultured in DMEM/Ham’s F-12 (1:1) (D6421; Sigma) under 5% CO2 and 37 °C with 10% fetal calf serum (F7524; Sigma) and 4 mM L-glutamine (G7513; Sigma). By incubating cells at 37 °C for 5 min with 0.1% trypsin and 0.02% EDTA, cells were detached and split 1:2. Cells were counted using an automated cell counter (Beckman Coulter) Vi-CELL XR. (All experiments were performed with one donor in three independent replicates).

### Senescence induction with doxorubicin

HDFs at passages 13–17 were seeded at 3500 cells/cm^2^. SIPS cells received the first dose of 150 nM doxorubicin one day and the second dose 4 days after seeding, one week after seeding the doxorubicin-containing culture media was changed to normal culture media and one week later these cells were used for the experiments. During this period from seeding to assessment, for quiescent cells media was changed regularly and for proliferating cells, 1:2 splitting was performed twice a week.

### SA-β-gal staining and BrdU incorporation

SA-β-gal staining was performed based on a standard protocol (Dimri et al. 1995). 10 images per well were taken (magnify x 100) and after randomization of images, the positive cells were counted blindly.

BrdU incorporation was performed using flow cytometry according to a standard protocol (Zhu 2012) and the percent of BrdU-positive cells was determined with CtyExpert software.

### qPCR

TRI Reagent (Sigma) was used to lyse the cells, and total RNA was isolated with Direct-zol RNA Miniprep (R2050; Zymo) according to the manufacturer’s instructions. cDNA was synthesized with High-Capacity cDNA Reverse Transcription Kit (500ng of total RNA, Applied Biosystems) and transcript levels were determined using the 5x HOT FIREPol® EvaGreen® qPCR Mix Plus with ROX (Solis BioDyne), specific primer pairs and a Rotor-Gene Q cycler (Qiagen) and the gene expression was normalized to GAPDH.

List of primers used for mRNA expression analyses:

GAPDH forward: CGACCACTTTGTCAAGCTCA, Reverse: TGTGAGGAGGGGAGATTCAG.

p21 forward: GGCGGCAGACCAGCATGACAGATT, Reverse: GCAGGGGGCGGCCAGGGTA.

Lamin B1 forward: AAGCAGCTGGAGTGGTTGTT, Reverse: TTGGATGCTCTTGGGGTTC.

ACTA2 forward: GTGAAGAAGAGGACAGCACTG, Reverse: CCCATTCCCACCATCACC.

FN1 forward: TGTCAGTCAAAGCAAGCCCG, Reverse: TTAGGACGCTCATAAGTGTCACCC.

Col1A1 forward: GCTCCGACCCTGCCGATGTG, Reverse: GGCTCCGGTGTGACTCGTGC.

### Conditioned media preparation

One week after doxo-treatment to induce senescence, the media was changed to DMEM/HAM’s F12 without serum. SIPS and proliferating cells were incubated for 48 h in this media, then supernatants were collected, centrifuged at 200 g for 5 min, and filtered through a 0.22 μm syringe filter.

### Collagen contraction assay

The wells of a 24 well-plate were precoated with 20% FCS and kept in the fridge overnight, after washing twice with PBS, the mixture of 600 µL NaOH 0.7 M, 600µL HEPES buffer, 1 × 10^6^ fibroblast cells in 1.2 mL 10X MEM and 9.6 mL type 1 collagen (4 mg/ml Collagen G from bovine calf serum, Merck) were prepared and placed in the wells (final cell number was 3,800 cells/well.). The collagen-embedded cells were maintained at 37 °C, in a 5% CO2 atmosphere for 1 h to allow for the formation of collagen fibers. The collagen lattices were then gently detached from the plate wells and resuspended in 1 mL of CM, collagen lattice diameter was measured at 0,1,2,3 days and the average of contraction was measured with ImageJ as the percentage of diameter change compared to the diameter at day 0.

### Immunofluorescence

Non-senescent HDF cells were seeded into 8 well µ-slides (80,826, Ibidi) with a split ratio of 1:2 and after attachment, cells were treated for 72 h with prepared CMs. After washing twice with PBS, cells were fixed with 4% paraformaldehyde (Sigma-Aldrich), permeabilized in 0.3% Triton X-100 plus 5% FCS (Sigma-Aldrich) and then blocked with 5% BSA for 1 h. Cells were incubated overnight with α-SMA antibody diluted 1:100 in 1% BSA in PBS (F3777, Sigma-Aldrich). Cells were then washed and incubated with DAPI (Thermo Fisher Scientific) diluted 1:50 for 10 min. Slides were imaged using a LAS X (Leica Microsystems, DMI8-CS) microscope at 20 magnification.

### Statistical analysis

GraphPad Prism 9.0.0 and R 3.2.0 were used for statistical analysis and graph generation. Data are expressed as the mean ± S.D. Significance levels were denoted as: *P < 0.05, **P < 0.01, ***P < 0.001 and ****p < 0.0001. Unpaired t-test for comparison of two groups and one-way ANOVA and two-way ANOVA for comparison of multiple groups were used as depicted in the figure legends.

## Results

### Doxorubicin induces stress-induced premature senescence in HDFs

Treatment of HDFs with two doses of 150nM doxorubicin with three days intervals between the two treatments induced senescence in HDFs. These SIPS cells were assessed for several senescence markers including:

### SA-β-gal activity

**Fig. 1:**
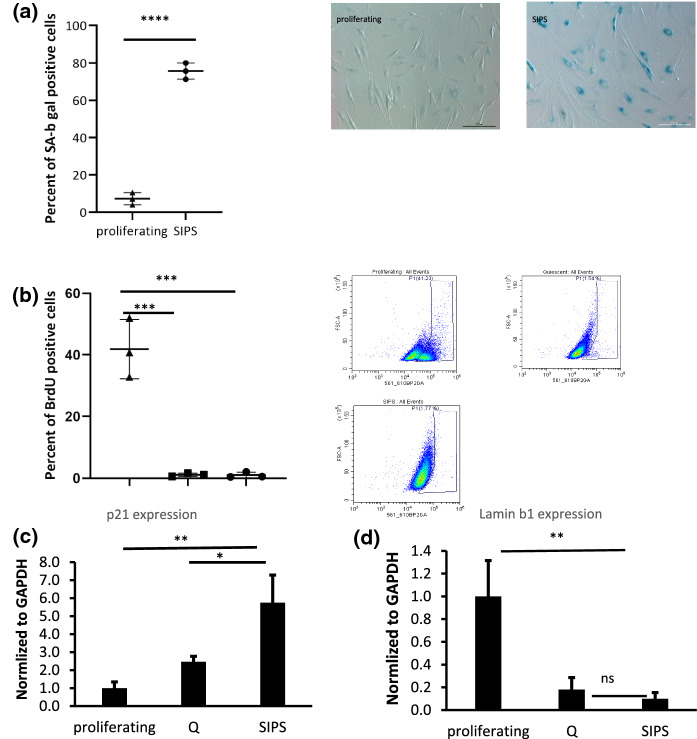
Characterization of senescence markers in doxorubicin-induced SIPS HDFs. HDFs were treated with two doses of 150 nM of doxorubicin and their senescent phenotype was assessed by **a** SA-β-gal staining (mean ± S.D.; n = 3, ****P ≤ 0.0001, unpaired *t*-test was used for data analyzing; scale bar, 200 μm), **b** BrdU incorporation (mean ± S.D.; n = 3, ***P ≤ 0.001, one way ANOVA followed with Dunnett’s multiple comparison test was used for data analyzing) and **c**, **d** qPCR for relative quantification of p21 and Lamin b1 gene expression. Non-senescent proliferating and quiescent cells were used as controls. (mean ± S.D.; n = 3, *P ≤ 0.05, **P ≤ 0.01, one-way ANOVA was used for data analysis)

Elevated SA-β-gal activity is a widely used and well-known senescence marker. The enzymatic activity, which is found in many normal cells under physiological conditions (pH 4.0–4.5), is significantly amplified in senescent cells at pH 6 as a result of increased lysosomal content (Herranz and Gil 2018). SIPS HDFs showed a significant increase in SA-β-gal activity with 75% of cells stained positive for this enzyme activity whereas only 7% of proliferating cells were positive (Fig. 1a).

### BrdU incorporation

The absence of BrdU incorporation, as a measurement of cell proliferation, is another marker for senescent cells (Adams 2009; Lämmermann et al. 2018). Only about 1% of SIPS cells were positive for BrdU in comparison with proliferating cells which were 42% positive. Quiescent cells (Q) were used as negative control which like SIPS cells were about 1% positive for BrdU (Fig. 1b). Thus, the experiment showed a significant difference between proliferating cells versus SIPS and Q cells.

### p21 and LMNB1 gene expression

The cell cycle inhibitor p21^Cip1^ (p21) is one of the major cell cycle regulators, as well as one of the most recognized and used markers for senescent cells (Wang et al. 2021). p21 expression was significantly upregulated in SIPS versus proliferating cells and also between SIPS and Q cells (Fig. 1c).

Another senescence marker is Lamin B1, which has been shown to decline in human fibroblasts induced to senesce by diverse stimuli (Freund et al. 2012; Wang et al. 2021). The expression of Lamin B1 (LMNB1) in SIPS was significantly lower than that in proliferating cells, but there were no significant differences between Q and SIPS cells (Fig. 1d).

## Profibrotic genes expression is elevated in senescent HDFs

**Fig. 2:**
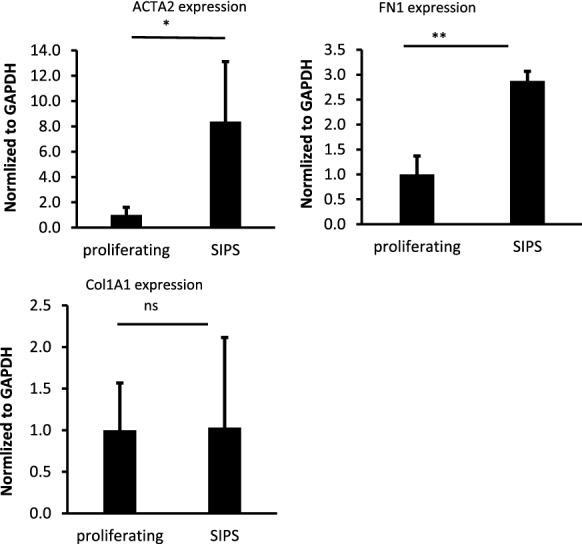
Fibrotic gene expression in senescent HDFs. The expression of **a** ACTA2, **b** FN1, and **c** COL1A1 was assessed in doxo-induced senescent cells compared to non-senescent proliferating cells. (mean ± S.D.; n = 3, *P ≤ 0.05, **P ≤ 0.01, unpaired student *t*-test was used for data analysis)

To find out the fibrotic potential of senescent HDFs, we evaluated some fibrosis-related gene expressions in SIPS cells and proliferating cells. These genes included ACTA2 (ɑ-smooth muscle actin), COL1A1 (collagen α1 type 1), and FN1 (fibronectin 1). The expression of ACTA2 is a key factor for myofibroblast differentiation, as well as a major contributor to contractile force production and stiffness of the ECM (Zeng et al. 2015; Hinz et al. 2012). FN1 is essential for collagen fibril assembly and COL1A1 is fibril-forming collagen (Zeng et al. 2015). According to our findings, the expression levels of ACTA2 were about 8-fold higher in SIPS cells compared to proliferating cells (Fig. 2a). Also, the expression of FN1 increased significantly in SIPS cells (Fig. 2b), but we could not detect any significant difference for COL1A1 when comparing SIPS versus proliferating cells (Fig. 2c).

## Conditioned media from SIPS cells does not induce a paracrine fibrotic response in non-senescent cells

**Fig. 3:**
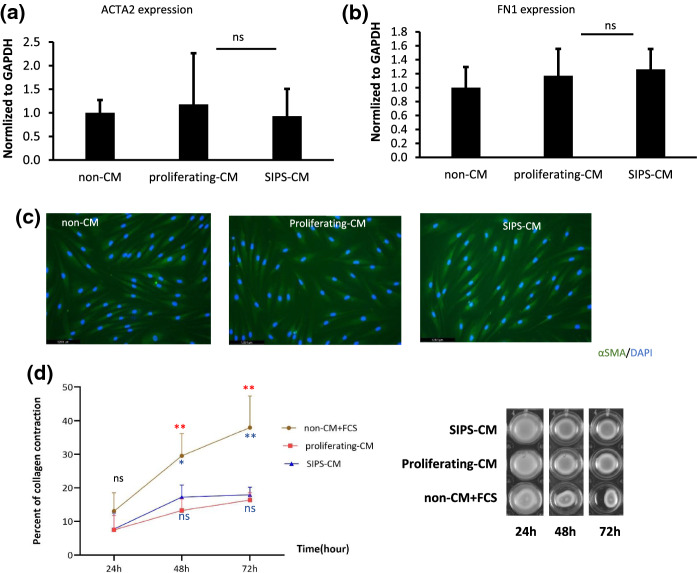
Fibrotic response of non-senescent HDFs to SIPS, proliferating and non-conditioned media. The fibrotic responses were assessed 72 h after treatment by qPCR for gene expression of **a** ACTA2 and **b** FN1(mean ± S.D.; n = 3, one-way ANOVA was used for data analysis), **c** immunostaining for αSMA (green) and DAPI (blue), **d** collagen contraction assay (mean ± S.D.; n = 6, *P ≤ 0.05, **P ≤ 0.01, two-way ANOVA followed with Tukey’s multiple comparisons test was used,  non-CM group versus proliferating-CM,  non-CM versus SIPS-CM)

As ACTA2 and FN1 expression increased in SIPS, we investigated whether conditioned media (CM) from these cells can induce a fibrotic response in non-senescent cells. This question was addressed by treating proliferating cells with conditioned media from SIPS cells (SIPS-CM), from proliferating cells (proliferating-CM), and non-conditioned media (non-CM) for 72 h and observing their fibrosis-related responses to this conditioned medium. qPCR results revealed that the expression of ACTA2 and FN1 did not change in SIPS-CM treated cells compared to cells treated with proliferating-CM and non-CM (Fig. 3a). Similarly, we did not see any significant difference in α-SMA intensity between the three groups after immunostaining. A collagen contraction assay was used to measure the contractile force of these cells. Although there was a significant difference between the group treated with culture media plus 10% FCS (positive control) and the two groups treated with SIPS-CM and proliferating-CM, no significant differences were observed between SIPS-CM and proliferating-CM (Fig. 3c).

## Discussion

In this study, we first confirmed the induction of cellular senescence in HDFs by doxorubicin. Doxorubicin is a chemotherapeutic drug that is used to treat several cancers by causing DNA damage that leads to the permanent exit of the cells from the cell cycle (Demaria et al. 2017). Here we used proliferating and quiescent cells as a control. Quiescent cells also exit the cell cycle, in this case, because of limitations in space for proliferation, but this cell-cycle arrest is reversible. Thus, after the cells are split, they can proliferate again in contrast to SIPS for which the cell cycle arrest is irreversible. In line with this, we could show that doxorubicin-induced senescent HDFs did not incorporate BrdU and showed increased SA-β-gal activity. Our study showed that p21 expression was significantly induced in SIPS cells compared to proliferating cells and quiescent cells, which further supports the validity of p21 as a senescent cell marker (Fig. 1c). As another marker of senescence, Lamin B1 expression was significantly lower in SIPS cells compared to proliferating cells. However, there was a trend, but no significant decrease in Lamin B1 expression between SIPS and quiescent cells. Thus, Lamin B1 expression could not discriminate between quiescent and senescent cells in our experimental set-up in contrast to other findings (Freund et al. 2012).

Many of the studies examining how senescent cells affect their surrounding microenvironment with their secretome are aimed at lung fibrosis, especially in the context of IPF. Many of these studies are based on the idea that senescent cells can alter their environment with SASP factors. The role of senescent lung fibroblast cells in IPF was studied in 2017 by Schafer and colleagues. According to their study, the SASP signaling of senescent IMR90 cells in IPF is profibrotic and senescent cells exert negative paracrine effects through SASP signaling. The expression of several fibrosis genes was elevated in non-senescent human IMR90 cells treated with media conditioned by senescent cells when compared to the control groups (Schafer et al. 2017). They concluded that cellular senescence at least partly is the mediator of IPF-related fibrosis. In our experiments, we used dermal fibroblast and doxorubicin to induce senescence. The expression levels of fibrosis-related genes (ACTA2, FN1) were significantly upregulated in senescent cells when compared to non-senescent cells. But by treating non-senescent cells with conditioned media of senescent cells, we could not observe a paracrine fibrotic response of their secretome, which is in contradict with the findings of Schafer et al. Although the causes of conflict observed in the two studies about fibroblast’s conditioned media on non-senescent cells are still unclear, a possible explanation may be due the heterogeneity of fibroblasts. Despite the shared function of fibroblasts in ECM synthesis in all organs to make connective tissue, we know they exhibit heterogeneity within and between tissues. In a comparison of several organs (skeletal muscle, heart, intestine, and bladder), fewer than one-fifth of fibroblast-enriched genes overlapped between them (Muhl et al. 2020). In addition, HDFs do not belong to the same lineage as IMR90. In this study, HDFs were obtained via abdominoplasty from human abdomens. Lung fibroblasts arise from endoderm during embryonic development, and they differ from dermal fibroblast progenitors which may come from either ectoderm or mesoderm. Connective tissue components are influenced by the origin of fibroblast populations (Plikus et al. 2021). These components could give them different functions in response to cell signaling pathways, especially to TGF-β and WNT signaling which are critical for fibrosis processing. In our study, the expression levels of ACTA2 and FN1 in non-senescent cells treated with CMs did not change. Furthermore, our immunofluorescence staining results for α-SMA in three groups also did not show significant differences. There were some cells in all groups that acquired myofibroblasts features, but the number of cells with these features was not significantly different between the groups (Fig. 3c). The last method we used for the evaluation of the fibrotic response of non-senescent HDFs to CM from SIPS was the collagen contraction assay. This assay has been extensively used for in vitro wound healing studies (Zhang et al. 2022). However, researchers believe this assay could reflect the situation of in vivo wound healing as well (Carlson and Longaker 2004). During fibrosis, fibroblasts differentiate into myofibroblasts and remodel the ECM and it has been demonstrated that the myofibroblasts found in fibrotic tissues have a stronger ability to contract collagen matrix than control fibroblasts in normal tissues (Zhang et al. 2022). There is a study from 2016 in which Midgley and colleagues showed that aged fibroblasts can transform into myofibroblasts less quickly than young fibroblasts in response to TGF-beta, which may explain the defect in wound healing in the elderly (Midgley et al. 2016). Since our study was about the fibrotic response of young fibroblasts to SIPS-CM, we hypothesized, that the contraction of the collagen lattice would increase in the SIPS-CM treated group but as we showed in Fig. 3d, we did not observe any significant difference between SIPS and proliferating-CM treated groups. Thus, in conclusion, despite the upregulation of fibrotic genes in SIPS HDFs, we could not find any evidence that the secretome of SIPS cells induces a paracrine response in non-senescent fibroblasts.
